# Risk Factors of Primary Dysmenorrhea in Female Adolescent Basketball Players Related to Dietary, Hormonal, and Immuno-Metabolic Factors and Disordered Eating Attitudes

**DOI:** 10.3390/nu17071190

**Published:** 2025-03-28

**Authors:** Małgorzata Mizgier, Barbara Więckowska, Veronica Sansoni, Amir Mohammad Malvandi, Grażyna Jarząbek-Bielecka, Michalina Drejza, Kinga Mruczyk, Angelika Cisek-Woźniak, Giovanni Lombardi

**Affiliations:** 1 Department of Sports Dietetics, Faculty of Health Sciences, Poznan University of Physical Education, 61-871 Poznan, Poland; 2 Department of Computer Science and Statistics, Poznan University of Medical Sciences, 60-806 Poznan, Poland; 3 Laboratory of Experimental Biochemistry and Advanced Diagnostics, I.R.C.C.S. Ospedale Galeazzi-Sant’Ambrogio, Via Cristina Belgioioso 173, 20157 Milano, Italy; veronica.sansoni@grupposandonato.it (V.S.); amirmohammad.malvandi@grupposandonato.it (A.M.M.); giovanni.lombardi@grupposandonato.it (G.L.); 4 Division of Developmental Gynaecology and Sexology, Department of Gynaecology, Poznan University of Medical Sciences, 61-758 Poznan, Poland; grajarz@tlen.pl; 5 Department of Obstetrics and Gynaecology, Cambridge University Hospitals NHS Foundation Trust, Cambridge CB2 0QQ, UK; michalina.drejza@gmail.com; 6 Department of Dietetics, Poznan University of Physical Education, 61-871 Poznan, Poland; k.mruczyk@awf-gorzow.edu.pl (K.M.); a.cisek@awf-gorzow.edu.pl (A.C.-W.); 7 Department of Athletics, Strength and Conditioning, Poznan University of Physical Education, 61-871 Poznan, Poland

**Keywords:** primary dysmenorrhea, menstruation, adolescent gynecology, nutrition, young female athletes, adolescent basketball players

## Abstract

**Background/Objectives:** Primary dysmenorrhea (PD) is a condition characterized by painful pelvic cramps onsetting shortly before menses and lasting for 3 days, negatively impacting the quality of life of young females. Further, menstrual cycle disorders are common in athletes. This study investigated differences in dietary habits, hormonal and immuno-metabolic parameters, and susceptibility to disordered eating attitudes (DEAs) between dysmenorrheic (D group) and non-dysmenorrheic (no-D group) young female basketball players. It also aimed to identify risk factors for PD, focusing on nutrition, anthropometric parameters, and biochemical markers. **Materials and Methods:** The study included 25 female basketball players (mean age: 16 years), categorized into D and no-D groups. Blood samples were analyzed for hormonal, metabolic, and inflammatory markers, including follicle-stimulating hormone, luteinizing hormone, total testosterone, androstenedione, dehydroepiandrosterone sulfate, estradiol, sex hormone-binding globulin, cortisol, prolactin, fasting glucose, fasting insulin, C-reactive protein, lipid profile, and 25-hydroxyvitamin D3. Dietary intake was assessed via a three-day food record, and DEA susceptibility was evaluated using the Eating Attitudes Test (EAT-26). Logistic regression identified independent PD risk factors. **Results:** The D group had significantly higher EAT-26 scores and prolactin and cortisol levels than the no-D group (*p* = 0.0284, *p* = 0.0108, *p* = 0.0035, respectively). Elevated prolactin, cortisol, and EAT-26 scores were associated with increased PD risk (OR = 1.75; OR = 1.02; OR = 1.14). **Conclusions:** Female basketball players with PD show higher prolactin and cortisol levels and greater DEA susceptibility. These factors may contribute to PD risk, warranting further research.

## 1. Introduction

### 1.1. Dysmenorrhea

Dysmenorrhea is a condition characterized by painful cramps during menstruation. The pain is most commonly localized in the lower abdomen but may also radiate to the lumbosacral region [1].

Regarding systemic symptoms, dysmenorrheic patients may experience both physical and psychological symptoms. Physical symptoms include headache, lethargy, fatigue, sleep disturbances (both hypersomnia and insomnia), breast tenderness, a heavy lower abdomen, back pain, myalgia, arthralgia, swollen legs, and gastrointestinal issues such as appetite changes, nausea, vomiting, and bloating. Psychological symptoms include mood disturbances, anxiety, depression, irritability, and nervousness [2,3].

### 1.2. Types of Dysmenorrhea

Dysmenorrhea is classified into primary and secondary dysmenorrhea. Primary dysmenorrhea (PD) is defined as pelvic pain occurring during menstruation without any coexisting gynecological pathology. It typically emerges within a year after menarche and is most intense during adolescence. Secondary dysmenorrhea, on the other hand, may occur after a period of painless menstrual cycles and is often associated with gynecological disorders such as pelvic inflammatory disease, congenital Müllerian anomalies, adnexal abnormalities, or conditions like endometriosis [4,5].

The estimated prevalence of PD among women of reproductive age ranges from 45% to 95%, with 2% to 29% of affected women experiencing severe pain [2,6,7].

### 1.3. Risk Factors for Dysmenorrhea

Known risk factors for dysmenorrhea include heavy menstrual bleeding, regular menstrual cycles, age below 30 years, coexisting premenstrual syndrome (PMS), low body mass index (BMI), and early menarche (before 12 years of age) [8].

### 1.4. Pathophysiology of Dysmenorrhea

The pathogenesis of dysmenorrhea is primarily related to increased intrauterine secretion of prostaglandins F2α and E2. These prostaglandins, synthesized through the arachidonic acid cascade and mediated by the cyclooxygenase pathway, enhance myometrial contractions and vasoconstriction, leading to uterine ischemia and the production of anaerobic metabolites. Consequently, this process results in the hypersensitization of pain fibers and the pelvic pain characteristic of dysmenorrhea. The synthesis of arachidonic acid is regulated by progesterone levels, which peak in the mid-luteal phase [2].

PD is considered to have an unclear etiology and may be influenced by immune, endocrine, environmental, and psychological factors [9,10]. Therefore, in dysmenorrhea-related research, potential psychological and emotional effects associated with eating disorders (EDs) should be considered. EDs are disorders of the psychiatric sphere characterized by abnormal eating habits that negatively affect an individual’s physical and mental health. The most common ED is binge eating disorder. The Eating Attitudes Test (EAT-26) is a widely used tool for assessing disordered eating attitudes (DEAs) due to its reliability and reproducibility in detecting EDs in the general population [10,11].

Notably, to date, no studies have examined whether DEAs increase the likelihood of PD in female athletes, including basketball players. Further research is needed to determine whether DEAs are more prevalent in dysmenorrheic female adolescent athletes, particularly basketball players, compared to their non-dysmenorrheic counterparts. Moreover, to the best of our knowledge, no studies have identified other PD risk factors among basketball players, including those related to diet. Previous research has primarily focused on the prevalence of dysmenorrhea in female athletes across various sports disciplines [12,13].

Recent studies indicate that physical activity modulates PD symptoms [14,15]. While earlier research reported a higher prevalence of dysmenorrhea among female athletes, including basketball players [12], a more recent study by Dusek et al. involving 34 runners, 10 soccer players, 18 basketball players, and 10 ballet dancers found that dysmenorrhea was nearly twice as infrequent in female athletes compared to the control group (*p* < 0.001) [13]. The latest findings suggest that targeted interventions, such as high-intensity interval training (HIIT) and probiotic supplementation, may effectively alleviate PD symptoms [15].

### 1.5. Study Objectives

Considering this background, our study aimed to compare young female basketball players affected by PD with those not affected by PD by investigating nutritional intake, hormonal and immune–metabolic parameters, and susceptibility to DEAs to identify differences between the groups.

Another objective was to determine risk factors for PD in young female basketball players, specifically those related to DEA predisposition and macronutrient and energy intake, as well as anthropometric, hormonal, and immuno-metabolic parameters. In the case of macronutrients and energy intake being identified as PD risk factors among basketball players, dietary modifications may be explored in future randomized interventional studies to alleviate PD-related pain in young female athletes.

## 2. Materials and Methods

### 2.1. Study Participants and Design

This study, conducted between 2021 and 2022, involved 25 female basketball players from the AZS AJP Gorzów Sports Club in Poland. The participants engaged in a weekly physical activity regimen consisting of five athletic training sessions and three strength training sessions, each lasting 1.5 h.

Caucasian girls were included in the study, using the convenience sampling method. The inclusion criteria required participants to be at least two years post-menarche; have regular menstrual cycles; be free from any diagnosed illnesses; and not to have consumed stimulants, dietary supplements, or medications, including contraceptives, for at least three months prior to the study, as these could have influenced the study outcomes.

The participants were divided into two groups based on the presence or absence of dysmenorrhea: a dysmenorrheic group (D group, *n* = 11) and a non-dysmenorrheic group (no-D group, *n =* 14).

All subjects underwent a medical evaluation during preventive examinations conducted by a specialist in adolescent gynecology. However, four participants, including one from the no-D group and three from the D group, did not consent to blood testing.

This cross-sectional study was conducted in accordance with the Declaration of Helsinki.

### 2.2. Medical Interview

During the medical interview, conducted as part of the diagnostic process for dysmenorrhea, data were collected regarding the regularity of menstruation; age at menarche; presence, location, and intensity of menstrual pain; as well as the use of medications, supplements, and dietary aids.

During the gynecological examination performed by a specialist in adolescent gynecology, pathological conditions indicative of secondary dysmenorrhea were excluded. The predominant symptom reported by the participants was lower abdominal pain, lasting for less than three days and rated between 8 and 9 points on a ten-point Visual Analogue Scale (VAS).

### 2.3. Anthropometric and Body Composition Assessment

All measurements were conducted after a 12 h fasting period. Anthropometric assessments included height, body mass, and waist circumference (WC). Body weight was measured to the nearest 0.1 kg using SECA 899 digital medical scales (SECA GmbH & Co. KG, Hamburg, Germany). Height was measured to the nearest millimeter using a SECA 217 stadiometer (SECA GmbH & Co. KG, Hamburg, Germany). Participants were instructed to remove their shoes and outer garments before measurement. WC was measured to the nearest 0.1 mm using a Gulick anthropometric tape (Country Technology, Inc., Gays Mills, WI, USA) between the rib margin’s lower border and the iliac crest’s upper border (WC-mid). Body mass index (BMI) was calculated according to the World Health Organization (WHO) guidelines for children aged 5–19 years [16].

Body composition was assessed using a Tanita MC 780 body composition analyzer (Tanita, Tokio, Japan), which employs bioelectrical impedance analysis (BIA). This model provides a measurement accuracy of 100 g for individual components, including adipose tissue. Fat mass (FM) measurements were expressed both as a percentage (%) and in kilograms (kg). A detailed description of this methodology has been previously published [17,18,19,20].

### 2.4. Nutritional Evaluation

Dietary habits were assessed by a qualified dietitian using a three-day food record method [21]. Participants recorded all consumed food and beverages over three consecutive days [22]. To estimate portion sizes, they referred to a photographic food atlas [23]. Parents were asked to assist their daughters in documenting dietary intake. The collected data were analyzed using the Aliant software (Cambridge Diagnostics, Anmarsoft, Gdańsk, Poland, 2018), and the daily food intake of each participant was compared with the recommended nutritional and energy requirements outlined by the Human Nutrition Standards of the National Food and Nutrition Institute [24]. The detailed methodology of dietary assessment has been described in previous studies [19].

### 2.5. Eating Behavior Assessment

Eating disorder-related behaviors were evaluated using the self-reported Eating Attitudes Test (EAT-26) questionnaire [10,11,19]. The EAT-26 is one of the most widely used tools for detecting eating disorders in the general population [10,11]. This questionnaire comprises 26 questions related to attitudes, beliefs, and behaviors associated with eating and perceptions of body shape and weight. The responses are categorized into subscales that facilitate the identification of disordered eating attitudes.

- The first subscale, Dieting, primarily reflects restrictive eating behaviors and body dissatisfaction.
- The second subscale, Bulimia and Food Preoccupation, encompasses bulimic tendencies and compulsive eating behaviors.
- The third subscale, Oral Control, assesses behaviors and beliefs related to self-imposed control over food intake, often linked to anorexic tendencies.

Participants responded to each question using a Likert scale: *always*, *usually*, *often*, *sometimes*, *rarely*, or *never*. In the first 25 questions, always, usually, and often responses were assigned scores of 3, 2, and 1 points, respectively. In question 26, the scoring was reversed, with *never* receiving 3 points, *rarely* 2 points, and *sometimes* 1 point. A cutoff score of 20 or higher indicated potential disordered eating attitudes (DEAs) [10,11,25,26,27].

### 2.6. Biochemical Analysis

Two 7.5 mL blood samples were collected by a certified nurse at the Central Research Laboratory of the Academy of Physical Education in Poznań, a branch in Gorzów, for eligible participants. Blood levels of luteinizing hormone (LH), follicle-stimulating hormone (FSH), sex hormone-binding globulin (SHBG), 17-β-estradiol (Estradiol), total testosterone (TT), dehydroepiandrosterone sulfate (DHEA-S), fasting insulin (FI), fasting glucose (FG), triglycerides (TGs), total cholesterol (TC), and high-density lipoprotein cholesterol (HDL-C) were measured in the morning during the follicular phase (days 3–5) while fasting.

Hormone levels and SHBG were determined using the electrochemiluminescence immunoassay (ECLIA) method (Elecsys; Roche Diagnostics GmbH, Mannheim, Germany). TC, HDL-C, and TG plasma levels were assessed using an enzymatic colorimetric method (Roche Diagnostics GmbH, Mannheim, Germany). Low-density lipoprotein cholesterol (LDL-C) concentration was calculated using Friedewald’s formula: LDL − C (mg/dL) = TC − (HDL − C + (TG/5)). Plasma glucose levels were determined via the hexokinase enzymatic method [28].

Homeostasis model assessment for insulin resistance (HOMA-IR) was calculated using the following formula: HOMA-IR = fasting insulin (mU/mL) × fasting glucose (mmol/L)/22.5 [29,30].

The biochemical analyses were conducted at the Central Laboratory of the Gynecology and Obstetrics Clinical Hospital of the Poznań University of Medical Sciences.

C-reactive protein (CRP), 25-hydroxyvitamin D3 (25-(OH)D_3_), and androstenedione (A) were analyzed at the Laboratory of Experimental Biochemistry, IRCCS Ospedale Galeazzi-Sant’ Ambrogio, in Milan, Italy. Blood samples were stored at −80 °C before transportation and analysis. 25-(OH)D_3_ was measured using mass spectrometry using a MassChrom^®^ kit (Chromsystems Instruments & Chemicals GmbH, Gräfelfing, Germany). This kit streamlines sample analysis using a 96-well filter-plate format. Initially, a fixed volume of internal standard was combined with a precipitation reagent and the sample, followed by a brief shaking period and centrifugation to achieve effective protein precipitation. The clear eluate was then injected into the LC-MS/MS system (Xevo TQ-S micro Triple Quadrupole Mass Spectrometer, Waters Corporation, Milford, MA, USA), where interfering matrix components were removed using an online trap column before the analytes were separated in a high-resolution analytical column via APCI in positive mode. Combined with isotopically labeled internal standards, this approach minimizes matrix effects and ensures robust, precise quantification of 25-OH-Vitamin D3. The concentration of each sample was quantified using a standardized human serum sample serial dilution. CRP and androstenedione were analyzed in duplicate using commercial ELISA kits (DRG International, Inc., Springfield, NJ, USA) and displayed a sensitivity of 0.1 mg/L and 0.021 ng/mL, respectively. Absorbance was read by a VICTOR^®^ X3 Multilabel Counter (Perkin Elmer Inc., Waltham, MA, USA), and results were calculated following the manufacturer’s instructions.

### 2.7. Statistical Analyses

This study was designed as a pilot study. Mean values, standard deviations (SDs), and medians with interquartile ranges (Q1; Q3) were calculated for each parameter to illustrate the effect sizes obtained. Additionally, the odds ratio (OR) with a 95% confidence interval (95% CI) was provided as another measure of effect size.

In the context of this study, the odds ratio with a 95% confidence interval is a statistical measure used to assess the strength and direction of the association between the presence or absence of dysmenorrhea (D group vs. no-D group) and a given parameter (e.g., age, BMI, hormone concentration, etc.).

- OR = 1: Indicates no association between the examined parameter and the occurrence of dysmenorrhea. The odds of experiencing dysmenorrhea are the same in both groups.
- OR > 1: Suggests an increased likelihood of dysmenorrhea as the parameter value increases. The higher the OR, the stronger the association.
- OR < 1: Suggests a decreased likelihood of dysmenorrhea as the parameter value increases. The lower the OR, the stronger the association.

The 95% confidence interval (95% CI) indicates the precision of the OR estimate. The wider the interval, the lower the precision. If the confidence interval includes the value of 1, the association is not statistically significant.

To compare groups, the following statistical tests were used: the Student’s *t*-test (*t*-st) for normally distributed data with equal variances, Cochran–Cox correction for the t-test when variances differed between groups, and the Mann–Whitney test (M-W) for data not following a normal distribution. Normality was assessed using the Shapiro–Wilk test, and variance equality was tested with the Fisher–Snedecor test. The odds ratio with its confidence interval was determined using logistic regression modeling.

All statistical analyses were conducted using PQStat v 1.8.6 (Poznań, Poland), with the level of statistical significance set at *p* < 0.05.

As this was a pilot study, we assume that the results will provide reference values useful for future research, including sample size determination. The G*Power v 3.1.9.6 program (developed by the University of Düsseldorf, open-source) was used to illustrate the method for determining the necessary sample size for future studies, applying *t*-tests for two independent groups and the Mann–Whitney test.

## 3. Results

The no-D group did not differ significantly from the D group in terms of age (16.1 ± 0.54 vs. 16 ± 0.55), height (171.2 ± 6.95 vs. 171.4 ± 8.35 cm), body mass (63.4 ± 6.17 vs. 61.1 ± 9.76 kg), body mass index (BMI) (21.6 ± 1.11 vs. 20.7 ± 1.9), waist circumference (WC) (69.6 ± 6.9 vs. 69.1 ± 12.75 cm), fat mass percentage (FM%) (23.9 ± 3.34% vs. 22.6 ± 5.49%), fat mass in kilograms (FM kg) (15.2 ± 3.09 kg vs. 14.2 ± 5.16 kg), or age at menarche (12.7 ± 0.9 years vs. 12.0 ± 1.2 years). However, the mean values for age, body mass, BMI, WC, FM, and age at menarche in the D group were lower than those in the no-D group (Table 1).

The odds ratio (OR) values suggest that the likelihood of dysmenorrhea occurrence is higher among younger women, those with lower BMI, and those with an earlier age of menarche (OR = 0.72, 95% CI 0.16–3.28; OR = 0.67, 95% CI 0.38–1.19; OR = 0.52, 95% CI 0.23–1.16, respectively). However, since the 95% CI values include 1, the associations are not statistically significant, and definitive conclusions cannot be drawn (Table 1).

The mean concentration of 25-(OH)D_3_ appeared to be lower in the D group compared to the no-D group (62.8 ± 23.82 vs. 69.6 ± 23.82 nmol/L). Conversely, the mean CRP serum concentrations were higher in the D group than in the no-D group (1.0 ± 0.6 vs. 0.9 ± 0.9 mg/L), but the difference was not statistically significant (Table 2).

Regarding metabolic markers, the average TGs, TC, and LDL (median values only) appeared to be higher. HDL appeared to be lower in the D group than in the no-D group; however, these differences were insignificant. Additionally, the median and average FG, FI, and HOMA-IR serum concentrations appeared to be higher in the no-D group than in the D group (Table 2).

The mean prolactin concentration in the no-D group was 12.2 ± 2.13 ng/mL; in the D group, it was 18.5 ± 6.50 ng/mL. The median prolactin concentration in the no-D group was 11.77 ng/mL (interquartile range: 10.88–13.87 ng/mL), whereas in the D group it was 16.45 ng/mL (interquartile range: 13.91–20.45 ng/mL). The difference in means and medians was statistically significant (*p =* 0.01), confirming that girls with dysmenorrhea had higher prolactin levels. OR = 1.75 indicates that a higher prolactin concentration is associated with a greater likelihood of dysmenorrhea occurrence. The 95% CI 1.03–2.97 does not include 1, confirming the statistical significance of the result (Table 3).

The mean cortisol concentration in the no-D group was 304.6 ± 85.6 nmol/L, while in the D group it was 433 ± 180.63 nmol/L. The median cortisol concentration in the no-D group was 307.10 nmol/L (interquartile range: 254.75–334.93 nmol/L), whereas in the D group it was 379.40 nmol/L (interquartile range: 358.30–418.05 nmol/L). The difference in means and medians was statistically significant (*p =* 0.0035), indicating that girls with dysmenorrhea had higher cortisol levels. Additionally, OR = 1.02 suggests a slight increase in the likelihood of dysmenorrhea with increasing cortisol concentration. The 95% CI 1.00–1.04 does not include 1, indicating statistical significance (Table 3).

Moreover, the mean values for estradiol and 17-(OH)P appeared to be higher in the no-D group than in the D group (121.8 ± 106.03 vs. 84.5 ± 49.63 pg/mL; 1.3 ± 0.77 vs. 1.2 ± 0.33 ng/mL, respectively). On the other hand, the mean serum concentrations of androgens (except TT), including DHEA-S, A, and SHBG levels, appeared to be higher in the D group than in the no-D group. However, the median concentration of TT was slightly higher in the D group (Table 3).

It was observed that energy, fat, carbohydrate, and protein intake appeared to be lower in the D group than in the no-D group. The most pronounced differences were in sucrose and saturated fat (SFA) intake. The no-D group had a higher intake of saccharose (51.9 ± 23.32 g vs. 36.1 ± 18.48 g; *p =* 0.0586) and SFAs (34.3 ± 10.98 g vs. 26.2 ± 8.05 g; *p =* 0.0539) (Table 4).

On the other hand, the mean EAT-26 total score was significantly higher in the D group than in the no-D group (9.2 ± 5.32 vs. 6.1 ± 4.99; *p =* 0.0284). Moreover, a higher EAT-26 score significantly increased the probability of dysmenorrhea (OR = 1.14, 95% CI 0.95–1.36, *p =* 0.0284) (Table 4).

### Required Sample Size in the Context of the Pilot Study

Based on the results for BMI, age at menarche, and HOMA-IR, the estimated sample sizes required for future studies are as follows:

- **BMI:** To detect an effect size of *d* = 0.59127, 46 participants per group would be needed (Student’s *t*-test for two independent groups, α = 0.05, statistical power = 0.8).
- **Age at menarche:** To detect an effect size of *d* = 0.695648, 39 participants per group would be required (Wilcoxon–Mann–Whitney test, α = 0.05, statistical power = 0.8).
- **HOMA-IR:** To detect an effect size of *d* = 0.8256093, 25 participants per group would be necessary (Student’s *t*-test for two independent groups, α= 0.05, statistical power = 0.8).

To illustrate the method used for sample size determination, we provide an example based on the HOMA-IR parameter. The estimation was conducted using the mean difference observed in the pilot study, where the mean ± SD values were as follows:

- **No-D group: 2.42 ± 0.59;**
- **D group: 1.89 ± 0.69 (Table 2).**

These values were automatically converted in the G*Power software 3.9.1.7 into a standardized effect size of Cohen’s d = 0.8256093. The following parameters were entered into the program: Cohen’s d effect size, statistical power = 0.8, significance level = 0.05, and balanced group allocation (N_2_/N_1_ = 1).

**Figure 1:**
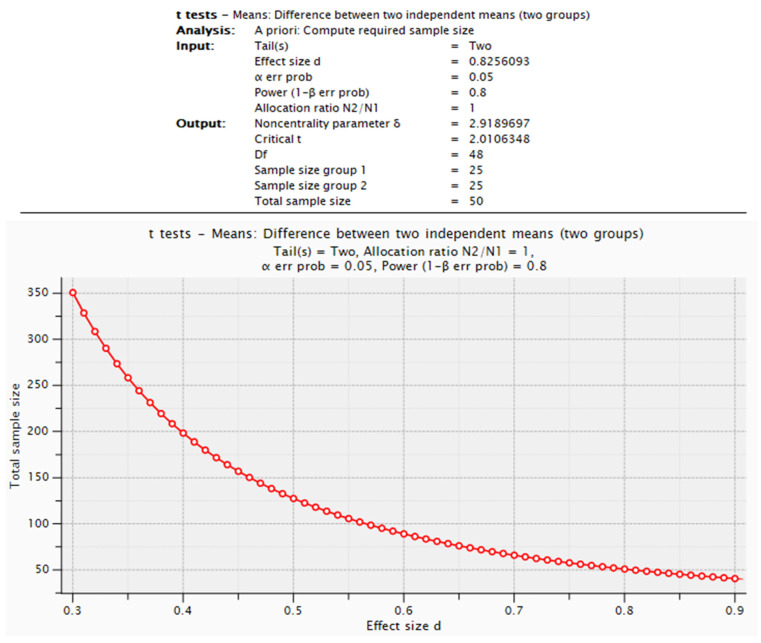
The graph illustrates the sample size estimation process.

As a result, the required sample size was determined to be *n* = 25 per group. The generated report and corresponding graph (Figure 1) visually represent the sample size estimation process. Additionally, the graph enables an analysis of how the required sample size would vary if the observed effect sizes were either smaller or larger than those obtained in this study.

Additionally, it is important to consider the dropout rate for realistic planning of future studies. In our study, the dropout rate was 4/25 (16%), which may be valuable information for other researchers when determining an appropriate sample size that accounts for potential data loss.

The method for calculating the required sample size considering this dropout rate $\left(n_{final}\right)$ is as follows:

$$n_{final}=\frac{n}{1-0.16}=\frac{25}{0.84}=30$$

## 4. Discussion

The maturation process in adolescent females is frequently associated with menstrual pain, which can significantly impact various aspects of quality of life, including academic performance, social relationships, and athletic achievements among young female athletes, including basketball players. Therefore, efforts should be directed toward improving the quality of life of adolescent girls and identifying factors contributing to menstrual pain, particularly modifiable factors (e.g., dietary habits) that individuals can influence to support therapeutic interventions.

Scientific research on dysmenorrhea in female basketball players remains limited, with only a few studies addressing this topic [12,13,15]. To bridge this research gap, our study aimed to compare adolescent female basketball players affected and unaffected by primary dysmenorrhea (PD) to identify differences between these groups. The specific factors of interest included energy and macronutrient intake, hormonal and immuno-metabolic parameters, and susceptibility to Disordered Eating Attitudes (DEAs). Given that previous studies conducted by our research team demonstrated an association between lifestyle factors—including dietary habits, predisposition to eating disorders, and specific anthropometric, hormonal, and immuno-metabolic parameters—and gynecological health in non-athletic young women [17,19,20,25,29,31,32,33,34], the present study sought to assess risk factors for PD in young female athletes, specifically basketball players. This research aimed to enhance the understanding of potential associations between PD and the investigated variables in young female athletes.

Our preliminary findings suggest that the likelihood of PD is higher in younger females (OR = 0.72; 95% CI 0.16–3.28), those with lower BMI (OR = 0.67; 95% CI 0.38–1.19), and those experiencing earlier menarche (OR = 0.52; 95% CI 0.23–1.16). Although none of these relationships reached statistical significance, all examined variables exhibited lower mean values in the PD group. Our results align with findings from studies on non-athletic females, which indicate that younger age, earlier menarche, and lower BMI increase the likelihood of dysmenorrhea [35]. Chauhan et al. identified a significant correlation between low BMI and dysmenorrhea, suggesting that improving the nutritional status of young females may reduce menstrual pain [36]. Other researchers have confirmed that dysmenorrhea prevalence declines with increasing age [37,38,39]. Additionally, Marques et al. reported that girls who experienced menarche before the age of 12 had nearly twice the risk of developing dysmenorrhea compared to those who reached menarche at 12 years or older [40].

Further findings indicate that serum 25-(OH)D_3_ concentrations were slightly lower in the PD group than in the non-PD group (68.8 ± 23.82 vs. 69.6 ± 23.82 ng/mL). In comparison, mean serum concentrations of the inflammatory marker C-reactive protein (CRP) were slightly higher in the PD group (1 ± 0.63 vs. 0.9 ± 0.9 mg/L). It is well established that vitamin D plays a crucial role in immune function and exerts anti-inflammatory effects [41]. Specifically, vitamin D modulates prostaglandin levels, which are involved in inflammation and calcium homeostasis, and may alleviate menstrual pain associated with dysmenorrhea [42]. A meta-analysis of randomized clinical trials demonstrated a significant association between reduced vitamin D intake and increased dysmenorrhea-related pain [43]. However, it should be outlined that, in our study, the measured 25-(OH)D_3_ values were virtually all above the adequacy limit (>50 nmol/L), and, therefore, no or minimal effects could be expected. Contrarily, it would be interesting to understand if an insufficiency/deficiency vitamin D status can modify PD symptoms. Additionally, Pakniat et al. found that supplementation with vitamin D, ginger, vitamin E, and fish oil—all possessing anti-inflammatory properties—significantly reduced dysmenorrhea severity [44]. Notably, an anti-inflammatory diet rich in antioxidants, nitrate-containing foods, unsaturated fatty acids (PUFAs and MUFAs), flavonoids, carotenoids, and vitamins C and E may also contribute to alleviating menstrual pain [45,46,47,48,49].

Regrettably, our study revealed that the PD group had a lower average intake of total energy and all macronutrients, including PUFAs, MUFAs, and fiber, which are found in vegetables and fruits and have anti-inflammatory effects. Previous research by our team on young women with polycystic ovary syndrome (PCOS) demonstrated that an anti-inflammatory diet reduces low-grade inflammation, oxidative stress, insulin resistance, and androgen concentrations [34]. Regular consumption of an anti-inflammatory diet is associated with beneficial changes in inflammatory markers, including CRP, glycemia, insulinemia, and lipidemia [50,51]. Consistently, our study found that the PD group, which consumed insufficient nutrients—including those with anti-inflammatory properties—exhibited higher lipid concentrations, including triglycerides (TGs), total cholesterol (TC), and low-density lipoprotein (LDL) cholesterol, while displaying lower high-density lipoprotein (HDL) cholesterol levels compared to the non-PD group. Interestingly, although mean serum concentrations of fasting glucose, insulin, and HOMA-IR were higher in the non-PD group, this group also exhibited higher saccharose (51.9 ± 23.32 g vs. 36.1 ± 18.48 g; *p =* 0.0586) and pro-inflammatory saturated fatty acid (SFA) intake (34.3 ± 10.98 g vs. 26.2 ± 8.05 g; *p =* 0.0539), which may partly explain these observations.

Moreover, our study demonstrated a significant difference in EAT-26 scores between the PD and non-PD groups. The mean EAT-26 score was significantly higher in the PD group (9.2 ± 5.32 vs. 6.09 ± 4.99, *p =* 0.0284), and an increased EAT-26 score was associated with a higher risk of PD (OR = 1.14, 95% CI 0.95–1.36, *p =* 0.0284), suggesting that greater susceptibility to DEAs may elevate PD risk. Although previous research has not established a direct link between dysmenorrhea and DEAs, we hypothesize that eating behaviors may influence menstrual pain. Eating disorders (EDs), characterized by abnormal eating patterns that negatively impact physical and mental health, are common among young females, with binge eating disorder being the most prevalent [9,10]. Dmitrović et al. identified binge eating behavior, depression, and poor sleep quality as risk factors for dysmenorrhea but did not elucidate the underlying mechanisms linking these factors [52]. Psychological stress, commonly observed in women with PD, can disrupt the endocrine system, leading to increased prostaglandin production, heightened myometrial contractions, and intensified dysmenorrhea symptoms [53]. Elevated cortisol levels have been implicated in stress-induced pain exacerbation in PD, as cortisol stimulates prostaglandin synthesis in the uterus, resulting in excessive uterine contractility, ischemia, and hypoxia [54]. Our findings support this, as serum cortisol concentrations were significantly higher in the PD group compared to the non-PD group (433 ± 180.63 vs. 304.2 ± 85.58 nmol/L, *p =* 0.0035), with OR = 1.02 indicating a slight increase in PD risk with rising cortisol levels.

Furthermore, the 95% CI (1.00–1.04) did not include 1, confirming statistical significance. These findings align with those of Zhou et al., who reported a similar relationship between stress, cortisol levels, and primary dysmenorrhea [55]. Additionally, we observed significantly higher serum prolactin concentrations in the PD group compared to the non-PD group (18.5 ± 6.5 vs. 12.2 ± 2.13 ng/mL, *p =* 0.0108). Increased prolactin levels were associated with an almost two-fold higher probability of PD (OR = 1.75; 95% CI 1.03–2.97; *p =* 0.0108). Previous studies indicate that plasma prolactin is significantly higher in females than males and is elevated in individuals with major depressive disorder [56]. Litschgi et al. also reported a potential correlation between hyperprolactinemia and severe PD, noting increased prolactin levels during the luteal phase in dysmenorrheic women [57]. Moreover, hyperprolactinemia has been shown to increase sensitivity to painful stimuli and to influence menstrual cycle regulation and ovulation, further supporting its involvement in PD pathophysiology [58].

It is also important to note that susceptibility to eating disorders (DEAs) may influence HPA axis function and the levels of hormones such as prolactin and cortisol. Previous studies suggest that individuals with abnormal eating patterns may exhibit altered stress hormone secretion, which could impact the severity of PD [59]. These mechanisms may involve neuroendocrine regulation disturbances, leading to HPA axis dysfunction and metabolic disorders.

In conclusion, our findings support the existence of a potential link between prolactin levels, cortisol levels, and susceptibility to DEAs and the occurrence of PD. Future studies should focus on identifying the mechanisms of interaction between these factors and their potential impact on therapeutic strategies for PD treatment.

### Strengths and Limitations

Several notable strengths of the present study should be highlighted. First, the potential underlying mechanisms linking primary dysmenorrhea (PD) to disordered eating attitudes (DEAs) have not been previously investigated in female basketball players. Second, this study is the first to evaluate the risk of dysmenorrhea in young female basketball players, considering dietary factors, DEA susceptibility, and concentrations of hormonal and immuno-metabolic markers. Third, it provides a novel comparison between dysmenorrheic and non-dysmenorrheic athletes.

However, certain limitations should be acknowledged. A key limitation was the limited number of studies investigating risk factors for dysmenorrhea in young female athletes, restricting the available literature for comparison. Thus, our research contributes to this field and highlights the need for further studies.

An additional limitation is represented by the lack of a non-athlete control group, which makes it difficult to determine whether the observed differences are specific to athletes or applicable to the general population.

Another limitation was the relatively small sample size, with an additional four participants declining blood sample collection. The relatively small sample size limits the generalizability of the findings. Nevertheless, it should be emphasized that this study is preliminary and was designed to guide future research in this area. Moreover, the observed dropout rate (4 out of 25) provides valuable insights for sample size estimation in subsequent studies conducted by our research team and other investigators.

## 5. Conclusions

In our study, young female basketball players experiencing dysmenorrhea exhibited higher EAT-26 scores, suggesting greater susceptibility to DEAs, along with elevated serum concentrations of prolactin and cortisol, compared to their non-dysmenorrheic counterparts. Furthermore, an increase in serum prolactin and cortisol levels, as well as higher EAT-26 scores, may be associated with an increased risk of PD. However, no significant differences in macronutrient and energy intake were observed between the groups.

Additionally, the greater predisposition to DEAs and the elevated cortisol and prolactin levels observed in the dysmenorrheic group may indicate heightened susceptibility to stress and depression in these young athletes. This, in turn, could negatively impact their gynecological health and athletic performance during adolescence. Therefore, targeted interventions should be implemented to improve the quality of life of these young female athletes.

In future research, a more comprehensive assessment of participants’ long-term dietary habits will be necessary to validate the dietary assessment method and ensure more consistent data. Further studies should consider the role of sleep patterns and psychological stress in the development of primary dysmenorrhea and its association with hormone levels and disordered eating behaviors. Moreover, future research should explore the influence of training intensity, physical activity levels, and psychological factors alongside hormonal, metabolic, and inflammatory markers to provide a more comprehensive understanding of dysmenorrhea in female athletes.

Further research involving a larger population is warranted to confirm and expand upon the findings of this preliminary study. Additionally, more extensive, multi-center studies with diverse populations will help validate these findings and explore the role of potential confounders.

Finally, while our study identified significant associations between elevated prolactin, cortisol levels, and premenstrual dysphoria (PD), it is important to emphasize that the cross-sectional design limits our ability to infer causality. We cannot determine whether these hormonal changes contribute to the development of PD or are secondary effects of the condition. Future longitudinal studies tracking hormonal profiles across menstrual cycles and interventional studies targeting these hormonal pathways are needed to elucidate the directionality and potential mechanisms underlying these associations. Additionally, larger, multi-center studies with diverse populations will help validate these findings and explore the role of possible confounders.

## Figures and Tables

**Table 1:** Basic characteristics of the studied groups: D group (girls with primary dysmenorrhea) and no-D group (girls without dysmenorrhea).

Parameters	Values	No-D Group (*n* = 11)	D Group (*n* = 14)	*p*	Test	OR [95% CI]
Age (years)	Mean ± SD	16 ± 0.54	16 ± 0.55	0.7026	M-W	0.72 [0.16, 3.28]
	Median [Q1; Q3]	16 [16; 16]	16 [16; 16]			
Body height (cm)	Mean ± SD	171.2 ± 6.95	171.4 ± 8.35	0.9379	*t*-st	1 [0.9, 1.12]
	Median [Q1; Q3]	173 [167; 175]	170.5 [166.75; 175]			
Body weight (kg)	Mean ± SD	63.4 ± 6.17	61.1 ± 9.76	0.4935	*t*-st	0.96 [0.87, 1.07]
	Median [Q1; Q3]	63.1 [58.35; 66.3]	61.25 [55.43; 64.85]			
BMI	Mean ± SD	21.6 ± 1.11	20.7 ± 1.9	0.1689	*t*-st	0.67 [0.38, 1.19]
	Median [Q1; Q3]	21.6 [20.7; 22.15]	20.75 [19.73; 21.95]			
FM (%)	Mean ± SD	23.9 ± 3.34	22.6 ± 5.49	0.5070	*t*-st	0.94 [0.78, 1.13]
	Median [Q1; Q3]	24.4 [22.85; 25.75]	22 [19.8; 26.03]			
FM (kg)	Mean ± SD	15.2 ± 3.09	14.2 ± 5.16	0.5445	*t*-st	0.94 [0.78, 1.14]
	Median [Q1; Q3]	15.2 [14.05; 16.6]	12.85 [11.7; 16.68]			
WC (cm)	Mean ± SD	69.6 ± 6.88	69.1 ± 12.75	0.8260	M-W	1 [0.92, 1.08]
	Median [Q1; Q3]	68 [66.5; 69.5]	68.5 [60.25; 73.5]			
Menarche (years)	Mean ± SD	12.7 ± 0.9	12 ± 1.18	0.0786	M-W	0.52 [0.23, 1.16]
	Median [Q1; Q3]	13 [12; 13]	12 [11; 12]			

Abbreviations: BMI—body mass index; FM—fat mass, WC—waist circumference. The analysis was conducted using an unpaired *t*-test (*t*-st) or Mann–Whitney test (M-W).

**Table 2:** Comparison between D group (girls with primary dysmenorrhea) and no-D group (girls without dysmenorrhea) in relation to inflammatory and metabolic measures.

Parameters	Values	No-D Group (*n* = 10)	D Group (*n* = 11)	*p*	Test	OR [95% CI]
CRP (mg/L)	Mean ± SD	0.9 ± 0.9	1 ± 0.63	0.9719	M-W	1.09 [0.34, 3.55]
	Median [Q1; Q3]	0.54 [0.46; 0.85]	0.68 [0.45; 1.34]			
25-(OH)D_3_ (nmol/L)	Mean ± SD	69.6 ± 23.82	62.8 ± 23.82	0.5035	M-W	0.97 [0.88, 1.07]
	Median [Q1; Q3]	61.77 [56.60; 73.75]	58.20 [47.83; 71.05]			
TC (mg/dL)	Mean ± SD	159.2 ± 35.65	162.5 ± 19.52	0.7885	*t*-st	1 [0.97, 1.04]
	Median [Q1; Q3]	153.4 [134.13; 182.18]	165.4 [153.65; 174.9]			
LDL (mg/dL)	Mean ± SD	80.7 ± 23.49	80 ± 14.22	0.9387	*t*-st	1 [0.95, 1.05]
	Median [Q1; Q3]	75.7 [63.33; 96.65]	82 [73.25; 87.85]			
HDL (mg/dL)	Mean ± SD	62.5 ± 14.04	63.3 ± 15.71	0.9072	*t*-st	1 [0.95, 1.07]
	Median [Q1; Q3]	62.15 [50.33; 71.1]	61.7 [48.7; 74.7]			
TGs (mg/dL)	Mean ± SD	76.1 ± 18.56	89.9 ± 31.05	0.2368	*t*-st	1.02 [0.99, 1.06]
	Median [Q1; Q3]	73.85 [65.4; 86.95]	74.8 [66.15; 118.55]			
FG (mg/dL)	Mean ± SD	93.3 ± 5.76	90.2 ± 7.69	0.3980	M-W	0.93 [0.8, 1.08]
	Median [Q1; Q3]	94.8 [89.08; 97.2]	92.2 [89.05; 94.35]			
FI (μU/mL)	Mean ± SD	10.4 ± 2.36	8.4 ± 2.68	0.1028	*t*-st	0.72 [0.49, 1.08]
	Median [Q1; Q3]	10.09 [8.06; 11.69]	7.64 [6.87; 10.6]			
Homa-IR	Mean ± SD	2.4 ± 0.59	1.9 ± 0.69	0.0868	*t*-st	0.26 [0.05, 1.29]
	Median [Q1; Q3]	2.43 [1.89; 2.76]	1.74 [1.45; 2.4]			

Abbreviations: CRP—C-reactive protein; TC—total cholesterol, LDL low-density lipoprotein cholesterol; HDL—high-density lipoprotein cholesterol; TGs—triglycerides, FG—fasting glucose [mg/dL]; FI—fasting insulin; HOMA-IR—Homeostatic Model Assessment of Insulin Resistance. The analysis was conducted using an unpaired *t*-test (*t*-st) or Mann–Whitney test (M-W).

**Table 3:** Comparison between D group (girls with primary dysmenorrhea) and no-D group (girls without dysmenorrhea) in relation to hormonal measures.

Parameters	Values	No-D Group (*n* = 10)	D Group (*n* = 11)	*p*	Test	OR [95% CI]
TT (ng/mL)	Mean ± SD	0.4 ± 0.17	0.4 ± 0.11	0.7057	*t*-st	0.28 [0, 139.88]
	Median [Q1; Q3]	0.3 [0.26; 0.52]	0.38 [0.28; 0.43]			
DHEA-S (µmol/L)	Mean ± SD	6.6 ± 3.06	6.9 ± 2.98	0.8098	*t*-st	1.04 [0.76, 1.42]
	Median [Q1; Q3]	6.54 [4.7; 7.9]	6.27 [4.87; 8.76]			
SHBG (nmol/L)	Mean ± SD	60.1 ± 13.77	76.3 ± 50.2	0.3277	C-C	1.01 [0.98, 1.04]
	Median [Q1; Q3]	60.23 [50.72; 66.83]	63.36 [44.69; 87.66]			
A (ng/mL)	Mean ± SD	1.7 ± 0.54	1.9 ± 0.45	0.4120	*t*-st	2.24 [0.35, 14.26]
	Median [Q1; Q3]	1.65 [1.31; 1.92]	2.03 [1.56; 2.23]			
FSH (mIU/mL)	Mean ± SD	5.1 ± 2.21	4.9 ± 2.18	0.8353	*t*-st	0.95 [0.63, 1.44]
	Median [Q1; Q3]	4.63 [3.55; 6.19]	5.18 [3.71; 6.13]			
LH (mIU/mL)	Mean ± SD	8.5 ± 6.09	9.9 ± 7.88	0.7513	M-W	1.03 [0.9, 1.17]
	Median [Q1; Q3]	6.72 [4.55; 10.52]	9.83 [4.21; 12.46]			
Estradiol (pg/mL)	Mean ± SD	121.8 ± 106.03	84.5 ± 49.63	0.6471	M-W	0.99 [0.98, 1.01]
	Median [Q1; Q3]	88.64 [41.82; 153.93]	73.78 [62.69; 113.76]			
Prolactin (ng/mL)	Mean ± SD	12.2 ± 2.13	18.5 ± 6.5	***0.0108***	C-C	1.75 [1.03, 2.97]
	Median [Q1; Q3]	11.77 [10.88; 13.87]	16.45 [13.91; 20.45]			
Cortisol (nmol/L)	Mean ± SD	304.2 ± 85.58	433 ± 180.63	***0.0035***	M-W	1.02 [1, 1.04]
	Median [Q1; Q3]	307.1 [254.75; 334.93]	379.4 [358.3; 418.05]			
17-OHP (ng/mL)	Mean ± SD	1.3 ± 0.77	1.2 ± 0.33	0.3416	M-W	0.78 [0.16, 3.66]
	Median [Q1; Q3]	0.94 [0.84; 1.25]	1.15 [1; 1.25]			

Abbreviations: TT—total testosterone; DHEA-S—dehydroepiandrosterone; SHBG—sex hormone-binding globulin; A—androstenedione; FSH—follicular-stimulating hormone; LH—luteinizing hormone. The analysis was conducted using an unpaired *t*-test (*t*-st) or Mann–Whitney test (M-W). All the *p*-values marked in bold and italics were statistically significant.

**Table 4:** Comparison between D group (girls with primary dysmenorrhea) and no-D group (girls without dysmenorrhea) in relation to energy, nutrient intake, and EAT-26 score.

Parameters	Values	No-D Group (*n* = 10)	D Group (*n* = 13)	*p*	Test	OR [95% CI]
Energy (kcal)	Mean ± SD	2310.9 ± 286.45	2101.1 ± 492.94	0.2452	t-st	1 [1, 1]
	Median [Q1; Q3]	2306.87 [2139.54; 2514.27]	1997 [1812.4; 2264]			
Protein (g)	Mean ± SD	93.3 ± 20.42	85.2 ± 19.23	0.3422	*t*-st	0.98 [0.94, 1.02]
	Media [Q1; Q3]	93.52 [76.91; 103.52]	79.41 [75.66; 100]			
Fat (g)	Mean ± SD	92.9 ± 29.36	75.8 ± 25.35	0.1497	*t*-st	0.98 [0.94, 1.01]
	Media [Q1; Q3]	91.02 [66.25; 113.07]	66 [63.88; 92.01]			
SFAs (g)	Mean ± SD	34.3 ± 10.98	26.2 ± 8.05	0.0539	(*t*-st)	0.9 [0.81, 1.01]
	Median [Q1; Q3]	32.63 [25.79; 40.14]	26.38 [21.65; 29.84]			
MUFAs (g)	Mean ± SD	28.7 ± 8.25	23.6 ± 8.1	0.1483	(*t*-st)	0.92 [0.83, 1.03]
	Median [Q1; Q3]	29.47 [21.45; 35.59]	24.04 [17; 28.1]			
PUFAs (g)	Mean ± SD	14 ± 8.78	10.8 ± 4.86	0.3521	(M-W)	0.93 [0.8, 1.07]
	Median [Q1; Q3]	11.17 [9.07; 15.18]	9.97 [8; 11.73]			
Cholesterol (mg)	Mean ± SD	366.8 ± 207.77	258.7 ± 103.68	0.1450	(M-W)	0.99 [0.99, 1]
	Median [Q1; Q3]	322.72 [251.34; 409.39]	225 [194; 341.4]			
Carbohydrates (g)	Mean ± SD	281.6 ± 36.44	276.4 ± 74.06	0.8280	(C-C)	1 [0.98, 1.01]
	Median [Q1; Q3]	271.92 [250.85; 306.82]	258 [209.9; 326.02]			
Fiber (g)	Mean ± SD	23.1 ± 6.6	20.2 ± 5.74	0.2810	(*t*-st)	0.92 [0.79, 1.07]
	Median [Q1; Q3]	22 [19.65; 22.52]	20 [18.01; 24.21]			
Saccharose (g)	Mean ± SD	51.9 ± 23.32	36.1 ± 18.48	0.0586	(M-W)	0.96 [0.92, 1.01]
	Median [Q1; Q3]	45.39 [42.03; 69.68]	33 [24; 41.63]			
EAT-26 score	Mean ± SD	6.1 ± 4.99	9.2 ± 5.32	0.0284	(M-W)	1.14 [0.95, 1.36]
	Median [Q1; Q3]	3 [3; 7.5]	7 [5; 12]			

Abbreviations: SFAs—saturated fatty acids; PUFAs—polyunsaturated-fatty acids; MUFAs—monounsaturated-fatty acids; EAT-26—Eating Attitudes Test total score. The analysis was conducted using an unpaired *t*-test (*t*-st) or Mann–Whitney test (M-W).

## Data Availability

The datasets generated and/or analyzed during the current study are not publicly available due to the data having been obtained without consent for sharing but are available from the corresponding author on reasonable request.
